# Longitudinal Impacts of Precision Greenness on Alzheimer’s Disease

**DOI:** 10.14283/jpad.2024.38

**Published:** 2024-02-16

**Authors:** S. C. Brown, W. W. Aitken, J. Lombard, A. Parrish, J. R. Dewald, R. Ma, S. Messinger, S. Liu, M. I. Nardi, T. Rundek, J. Szapocznik

**Affiliations:** 1 Department of Public Health Sciences, University of Miami Miller School of Medicine, Miami, FL USA; 2 University of Miami School of Architecture, Coral Gables, FL USA; 3 University of Miami Libraries, Coral Gables, FL USA; 4 Biostatistics Collaboration and Consulting Core, Division of Biostatistics, Department of Public Health Sciences, University of Miami Miller School of Medicine, Miami, FL USA; 5 Miami-Dade County Department of Parks, Recreation and Open Spaces (MDPROS), Miami, FL USA; 6 Evelyn F. McKnight Brain Institute, Department of Neurology, University of Miami Miller School of Medicine, Miami, FL USA; 7 University of Miami Clinical and Translational Science Institute, Miami, FL USA; 8 University of Miami Built Environment, Behavior, and Health Research Group, University of Miami Miller School of Medicine, 1120 NW 14th Street, Suite #1065, Miami, FL 33136 USA

**Keywords:** Alzheimer’s disease, prevention, environment, precision, greenness, NDVI

## Abstract

**Background:**

The potential for greenness as a novel protective factor for Alzheimer’s disease (AD) requires further exploration.

**Objectives:**

This study assesses prospectively and longitudinally the association between precision greenness - greenness measured at the micro-environmental level, defined as the Census block - and AD incidence.

**Design:**

Older adults living in consistently high greenness Census blocks across 2011 and 2016 were compared to those living in consistently low greenness blocks on AD incidence during 2012–2016.

**Setting:**

Miami-Dade County, Florida, USA.

**Participants:**

230,738 U.S. Medicare beneficiaries.

**Measurements:**

U.S. Centers for Medicare and Medicaid Services Chronic Condition Algorithm for AD based on ICD-9 codes, Normalized Difference Vegetation Index, age, sex, race/ethnicity, neighborhood income, and walkability.

**Results:**

Older adults living in the consistently high greenness tertile, compared to those in the consistently low greenness tertile, had 16% lower odds of AD incidence (OR=0.84, 95% CI: 0.76–0.94, p=0.0014), adjusting for age, sex, race/ethnicity, and neighborhood income. Age, neighborhood income and walkability moderated greenness’ relationship to odds of AD incidence, such that younger ages (65–74), lower-income, and non-car dependent neighborhoods may benefit most from high greenness.

**Conclusions:**

High greenness, compared to low greenness, is associated with lower 5-year AD incidence. Residents who are younger and/or who reside in lower-income, walkable neighborhoods may benefit the most from high greenness. These findings suggest that consistently high greenness at the Census block-level, may be associated with reduced odds of AD incidence at a population level.

## Introduction

Alzheimer’s disease (AD) – a leading cause of dementia worldwide – increasingly contributes to excess morbidity and mortality (1). Much research is being directed at prevention strategies to reduce the burden of AD across the globe (2). Most of this work focuses on individually-oriented interventions (1), such as improvements in modifiable factors like diet and exercise. Epidemiological research on AD has reported on modifiable risk factors including obesity (3), tobacco use (4), psychosocial stress and sleep (5). Further, the Finnish Geriatric Intervention Study to Prevent Cognitive Impairment and Disability (FINGER), a randomized clinical trial, showed a causal link between modifiable risk factors such as diet or exercise and cognitive functioning among older adults (6).

Beyond individual-level modifiable risk factors, environmental factors contribute to AD risk (7, 8). In particular, high levels of greenness – such as trees, shrubs, or other ground cover – has been shown to be cross-sectionally associated with reduced rates of AD (9, 10), and with risk factors for AD including cardiometabolic and cardiovascular disease (11–13). In addition, longitudinal research has linked greenness to lower AD mortality (14). More broadly, higher greenness has been associated with reduced risk for Parkinson’s disease, depression, and stroke, further reinforcing that greenness’ health impacts extend beyond a single chronic condition or a single diagnostic category (10, 15–17).

Greenness has been linked to lower levels of stress (18), increased physical activity, increased social connectedness (19), improved sleep (20), and attenuated negative impacts of air pollution (21), all of which could be mechanisms underlying greenness’ relationship to AD (6, 22–26). Accordingly, greenness may reduce odds of AD via one or more of these pathways.

Studies investigating the relationship of greenness — using the Normalized Difference Vegetation Index (NDVI, an objective measure of greenness using satellite imagery) (27) — to AD have used varying geographic units of analysis from the participant’s residence, ranging from 100 to 500 meter buffer sizes (14, 15). A 13-year longitudinal study examining the impact of a 500-meter residential greenness buffer revealed a 5% reduced risk of mortality due to AD (14). A case-control study examining the impact of a 100 meter greenness buffer on risk for AD did not find an association (15). However, our cross-sectional study of the relationship of greenness to AD at the Census block level found that Medicare beneficiaries residing in the highest tertile of greenness, when compared to those in the lowest tertile of greenness, had 20% reduced odds of AD, adjusting for neighborhood income and individual age, sex, and race/ethnicity (9, 10). It is noteworthy that our research found strong effects of Census block level greenness on AD, while Yuchi et al. using a similar size geographic area of 100 meter buffer, did not (15). Our team’s moderator studies found that age moderated the greenness to AD relationship (9), while neighborhood income was a marginally significant moderator of the greenness to AD relationship (10).

The current study builds on our prior research by examining longitudinally the impact of precision greenness measured at the micro-environmental level, defined as the Census block, on AD risk. The focus on the Census block in which the elder resides, adds precision to our understanding of the greenness to AD relationship. In this study we examine the longitudinal relationship between levels of greenness and AD incidence among older adults in Miami-Dade County, Florida, USA. Specifically, we investigate the relationship of consistently high versus consistently low greenness at the Census block level to the odds of AD incidence. We hypothesize that consistently high greenness blocks, when compared to consistently low greenness blocks, each in 2011 and 2016, will be associated with reduced odds for AD incidence from 2012 to 2016. If greenness at the Census block is associated with reduced AD incidence, this could enable greater precision in developing greenness prevention strategies. Such an approach could be a strong complement to existing individual-level prevention interventions. Additionally, to explore which populations may profit the most from high greenness, we evaluate the moderating effect of age, sex, neighborhood income and walkability as potential moderators of the greenness to AD relationship. We investigate walkability as a potential moderator because our prior research linked walkability to physical activity (28, 29), a protective factor for AD (6).

## Methods

### Design

A prospective, longitudinal design compared Medicare beneficiaries in Miami-Dade County living in two types of naturally occurring Census block conditions based on greenness across 2011 and 2016 (consistently low or Low-Low, and consistently high or High-High blocks). To determine levels (low, middle, high) of greenness, all Census blocks where Medicare beneficiaries reside were assessed for greenness level in each of 2011 and 2016. All greenness scores from blocks in which beneficiaries lived in 2011 and 2016 were used to calculate tertiles. The threshold established for the lower and upper tertiles were used to label blocks. Low-Low blocks were consistently in the low tertile in both 2011 and 2016, and High-High blocks were consistently in the high tertile in 2011 and 2016.

This study tests whether the immediate geographic area surrounding the person’s home, the Census block, is associated with reduced odds of AD incidence. Hence, in our use of “micro-environment” we refer to the block in which the elder resides (not time-space activity as has been used in other studies (30)). Census blocks are identified through the 9-digit ZIP code (i.e., ZIP+4). According to the US Census Bureau, “Census blocks, the smallest geographic area for which the Bureau of the Census collects and tabulates decennial census data, are formed by streets, roads, railroads, streams and other bodies of water, other visible physical and cultural features, and the legal boundaries shown on Census Bureau maps” (31). To illustrate the dimension of a Census block identified by a ZIP+4, it is useful to note that Census blocks are contained within Census block groups, which in turn are contained within Census tracts, which in turn are likely to be contained within ZIP codes, defined with 5 digits only. In urban areas, the Census block is typically comprised of a single city block bounded on four sides by streets and avenues.

### Study Population

**Figure 1:**
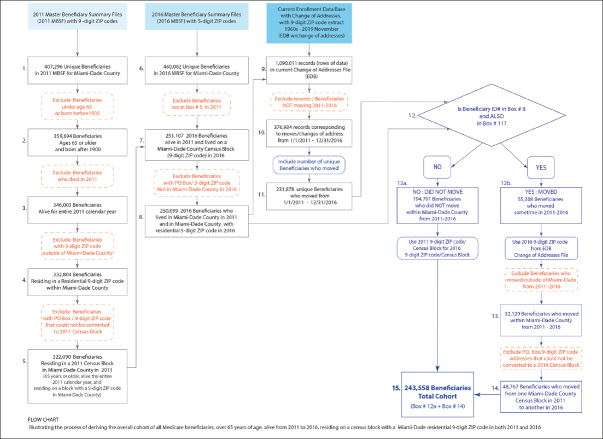
Derivation of the sample. The left column depicts initial sample of Medicare beneficiaries for 2011 and the exclusions to identify beneficiaries residing in a Census block in 2011, ages 65 and older, alive throughout 2011

The final study sample was derived in stages (See Figure 1). First, a cohort was identified comprised of all Medicare Beneficiaries 65 years or older, alive at the end of 2011 and at any time in 2016, who resided on a Census block with a Miami-Dade residential ZIP+4 (9-digit zip code) in both 2011 and 2016. To identify those who lived in a Census block, ZIP+4 data from CMS data were linked to a Census block for each beneficiary separately for 2011 and 2016, using GeoLytics ZIP+4 software (GeoLytics, Inc), which provides the area centroid of the ZIP+4 with latitude and longitude coordinates, and assigns the corresponding Census block and block group identification numbers. Specifically, starting with the 2011 CMS Master Beneficiary Summary File for Miami-Dade County, of 407,296 unique Medicare beneficiaries, the following exclusions were made. First, beneficiaries who were younger than 65 or born before 1900 were excluded (n=47,602), as were those who died at any time during 2011 (n=13,691), those whose residence could not be matched to either a ZIP+4 (n=13,199) or Census block (n=10,714), those who could not be matched to the 2016 CMS Master Beneficiary Summary File (n=70,983; possibly because they had moved out of the study area or had died), and those whose residence did not have 5-digit ZIP code information in 2016 (n=1,008). Finally, based on the Medicare Beneficiary Enrollment DataBase, we excluded beneficiaries who did not consistently reside in a Miami-Dade Census block during the entire 2011 to 2016 time period (n=6,541).

The second column from the left indicates exclusions to identify Medicare beneficiaries in 2011 and 2016. The third column identifies movers between 2011 and 2016, inclusive, on the basis of ZIP+4. The final column on the right, brings together non-movers with movers who remained within Miami-Dade County.

**Figure 2:**
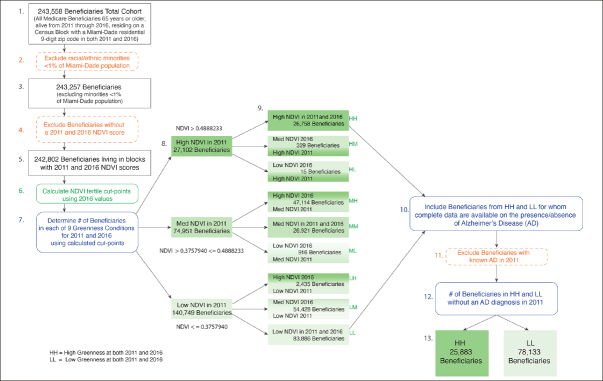
The residential ZIP+4 Census block of beneficiaries are scored for NDVI in 2011 and 2016

Next, these beneficiaries were linked to the 2011 and 2016 NDVI data (See Figure 2). NDVI scores were not available for 455 Medicare beneficiaries. This resulted in a cohort of 242,802 Medicare beneficiaries, aged 65 years and older who lived in Miami-Dade County (based on Medicare Beneficiary Enrollment DataBase) in 2011 and 2016. We then categorized beneficiaries into 9 subgroups based on NDVI tertiles in 2011 and 2016. Finally, we excluded 11,964 beneficiaries who already had AD in 2011, resulting in a final sample for this analysis of 230,738 beneficiaries. The study focus conditions were derived as Low-Low (n=78,133) and High-High (n=25,883). This study was approved by the University of Miami’s Institutional Review Board and CMS Privacy Board.

Census blocks are categorized according to the greenness tertile in which the beneficiaries resided in 2011 and 2016. Census blocks of beneficiaries living in the two study conditions (Low-Low and High-High) are identified.

### Variables/Measures

Centers for Medicare and Medicaid Services (CMS) Data: Alzheimer’s disease (AD) determination was made using the Chronic Conditions Data Warehouse algorithm (from the Chronic Conditions segment) for AD, from the U.S. Centers for Medicare and Medicaid Services (CMS). 2011 CMS data were obtained in 2013, and 2016 CMS data were obtained in 2020 (32). Specifically, the CMS chronic conditions algorithm for AD defines the presence/absence of AD using ICD9 codes for DX331.0 (any diagnosis for the claim, across claim types). The incidence of a new AD condition from 2012 through 2016 was used as the outcome in the present study.

Location. The CMS’ Master Beneficiary Summary File provided person-level data for location (ZIP +4 for 2011; ZIP+4 was not available in the CMS 2016 Master Beneficiary Summary File), health outcomes, age, sex and race/ethnicity. CMS’ Enrollment Database obtained in 2020 provided the history of known ZIP+4s, based on the “Beneficiary Residence Change Date» and “Beneficiary Mailing Contact Zip.» The Enrollment Database was used to determine who moved from 2011 and 2016. For non-movers, the ZIP+4 of 2011 was applied to 2016. For movers, the Enrollment Database provided the ZIP+4 for 2016.

Normalized Difference Vegetation Index (NDVI). NDVI has been established as a well-validated measure of neighborhood greenness for use in epidemiological research (12, 33). NDVI, a commonly used indicator of vegetation greenness, is correlated with specific physical properties of vegetation canopy such as leaf area index, fractional vegetation cover and vegetation condition (33). Many previous studies investigating the relationship between greenness and health have used NDVI (34–37). NDVI is computed by subtracting the reflectance of the red band from the reflectance of the near-infrared band (see Equation 1) (38). In areas with high levels of healthy vegetation, more red light tends to be absorbed for use in photosynthesis, while in areas with low levels of healthy vegetation less red light is absorbed and proportionally more red light is reflected (34). This study relies on one Landsat 5 image acquired on November 11, 2011 and one Landsat 8 image acquired on October 22, 2016. These images were obtained from the United States Geological Survey’s Earth Resources Observation and Science (EROS) Science Processing Architecture (ESPA) On Demand Interface (33, 34, 39). To avoid the need for additional atmospheric corrections, for both Landsat images Level 2 data were obtained (for surface reflectance, NDVI spectral index, and the pixel QA band). Cloud and cloud-shadow pixels were removed from the images using the pixel QA band (40, 41). Mean NDVI values were derived for each of the 23,856 Miami-Dade County Census blocks on which Medicare beneficiaries resided for both 2011 and 2016, resulting in a total of 47,712 NDVI values. These 47,712 NDVI values across 2011 and 2016, in turn, were categorized into three tertiles to create meaningful exposure categories of block-level greenness: the Low greenness tertile had an upper threshold of 0.376; the High greenness tertile had a lower threshold of 0.489.

Equation 1 $${\rm{NDVI}} = {{{\rm{NIR}} - {\mathop{\rm Red}\nolimits}} \over {{\rm{NIR}} + {\rm{Red}}}}$$

Covariates were individual age, sex, race/ethnicity, and neighborhood income, all of which have been linked to AD in prior research (1, 42–44). US Census Bureau 2011 data provided neighborhood median household income, as a continuous variable measured in thousands of dollars, at the level of the Census block group.

#### Moderators

We examined age (young old-65–74; old old-75 and over), sex, neighborhood income (median split at $41,100) and walkability (Walk Scores - 0–49 Car Dependent, 50–100 Non-Car Dependent) as potential moderators of the impact of greenness on AD incidence. We were unable to examine race/ethnicity as a moderator due to small sample size for Blacks (<3%) for the Low-Low condition.

#### Walkability

A literature review was performed using the electronic database PubMed (https://pubmed.ncbi.nlm.nih.gov/). Key terms were “Alzheimer’s disease” and “walkability”. These terms yielded 4 relevant abstracts, but none explicitly examined the association between walkability and AD. Walk Score® was used as a measure of walkability, which awards points based on distance to the nearest destination of each type (e.g., retail, recreational) using multiple data sources (e.g., Google, OpenStreetMap) (45, 46). Points are summed and normalized to produce a score of 0–100. Reliability and validity are acceptable (45–47). We obtained Walk Score in 2014 from Walkscore.com, which had been calculated in 2013 for the centroid of every Census block in Miami-Dade County. Because Walk Score does not archive data for prior years, we linked the participant’s location in 2011 to the 2013 Walk Score for their Census block.

### Statistical Analysis

All analyses were conducted using SAS version 9.4. Univariate descriptive analyses, found in Table 1, were conducted to describe the pattern of individual sociodemographics (age, sex, race/ethnicity), Census block group household income, NDVI, Walk Score, and AD incidence during the study period, first for the overall study sample, and then separately, for the Low-Low (LL) and High-High (HH) greenness categories.

**Table 1:** Descriptive statistics for the overall sample and by Census block Greenness Level (i.e., High-High and Low-Low greenness tertile conditions in 2011 and 2016, respectively)

**VARIABLE**	**OVERALL SAMPLE**			**HIGH-HIGH GREENNESS CONDITION**			**LOW-LOW GREENNESS CONDITION**			**HIGH-HIGH vs LOW-LOW**					
	**(All NDVI)**			**(Highest NDVI Tertile in 2011; Highest NDVI Tertile in 2016)**			**(Lowest NDVI Tertile in 2011; Lowest NDVI Tertile in 2016)**								
	**N (%)**	**Mean (SD)**		**N (%)**	**Mean (SD)**		**N (%)**	**Mean (SD)**		**T test**	**P-value**	**T test**	**P-value**	**T test/χ2 test**	**P-value**
		**2011**	**2016**		**2011**	**2016**		**2011**	**2016**	**2011**		**2016**		**2011–2016**	
N (Beneficiaries)	230,738 (100.00%)	---	---	25,883 (11.22%)	---	---	78,133 (33.86%)	---	---	---	---	---	---	---	---
Main Predictor: NDVI	---	0.35 (0.12)	0.43 (0.12)	---	0.55 (0.05)	0.62 (0.06)	---	0.23 (0.06)	0.29 (0.06)	809.17	<.0001	786.35	<.0001	---	---
Individual Sociodemographics:															
Age	---	74.02 (6.85)		---	73.50 (6.86)		---	74.35 (6.94)		---	---	---	---	−17.23	<.0001
Age ≥ 75 yrs (% Yes)	41.52%	---		38.08%	---		43.39%	---		---	---	---	---	224.38	<.0001
Gender (% Female)	57.77%	---		56.00%	---		58.18%	---		---	---	---	---	37.92	<.0001
Race/Ethnicity (RTI):										---	---	---	---	15216	<.0001
Hispanic (%)	66.88%	---		41.37%	---		81.21%	---							
Non-Hispanic White (%)	21.16%	---		44.35%	---		15.91%	---							
Black (%)	11.96%	---		14.28%	---		2.87%	---							
Neighborhood Median Household Income (Tens of thousands of dollars)	---	5.18 (3.06)		---	7.95 (4.49)		---	4.18 (2.26)		---	---	---	---	129.67	<.0001
Walkscore (2 levels)										---	---	---	---	14762	<.0001
Car-Dependent (1–49) (%)	36.42%	---		60.82%	---		20.65%	---							
Non-Car-Dependent (50–100) (%)	63.58%	---		39.18%	---		79.35%	---							
Alzheimer’s Disease:															
Development of Alzheimer’s Disease New Cases	4.86%	---		3.81%	---		6.04%	---		---	---	---	---	186.42	<.0001

To test the hypothesis that beneficiaries living in High-High greenness conditions is associated with reduced odds of AD incidence from 2012 to 2016, compared to beneficiaries living in Low-Low greenness conditions, we fit a multi-variable Generalized Estimating Equations (GEE) model with exchangeable correlation structure. This approach accounts for within Census block group correlation in estimates of variability used in tests of significance, and estimates population-averaged effects while adjusting for potential covariates. The GEE models were fit using PROC GENMOD to compare the relationship between HH versus LL conditions and the binary outcome of having a new AD diagnosis between 2012 and 2016, adjusting for age, sex, race/ethnicity, and neighborhood income and Walk Score.

Planned post-analyses explored the following potential moderators of the greenness to AD relationship: individual age, sex, neighborhood income and walkability. For the models exploring potential moderators of the greenness to AD relationship, cross-level interactions between greenness and the moderator of interest were examined in relation to AD incidence, adjusting for covariates. To maximize model fit, we assessed a series of candidate GEE models with different combinations of interaction terms between greenness and the moderator of interest. The Quasi-Likelihood Information Criterion with Correction for working Correlation (QICu) was the model selection criteria for the Generalized Estimating Equations (GEE) approach. The model with the lowest QICu value was selected from the candidate GEE models as the best-fit model, where the main effect of Greenness condition (HH vs LL), and the interaction terms of greenness with age (young old-65–74; old old-75 and over), neighborhood income (median split at $41,100) and walkability (Walk Scores – 0–49 Car Dependent, 50–100 Non-Car Dependent) were included, while adjusting for covariates.

## Results

The demographic characteristics for the overall study sample closely resemble those of all adults ages 65 and older for Miami-Dade County for this time period – which the 2013 U.S. Census estimated as 58% female, 68% Hispanic, 19% non-Hispanic White, and 13% Black. (48) Detailed descriptive statistics for the current sample are provided in Table 1. When comparing beneficiaries in the High-High greenness condition to beneficiaries in the Low-Low greenness condition, beneficiaries in the High-High condition were statistically significantly more likely to be younger, male, non-Hispanic Whites (44% vs. 16%), have higher median household income ($79,500 vs. $41,800); and less likely to be Hispanic (41% vs. 81%) and to have a high Walk Score (42 vs. 62). All ps<.0001.

### High-High vs. Low-Low conditions

The results of the multivariable analyses compared beneficiaries in the High-High greenness condition to those in the Low-Low greenness condition. Those in the High-High condition had a 16% lower odds of new AD diagnosis (OR=0.84; 95% CI: 0.76,0.93; p<.002), adjusting for individual age, sex, race/ethnicity, neighborhood income. Because physical activity also has been found to reduce risk for AD, (6) we conducted a post-hoc analysis further adjusting for Walk Score. However, there were no statistically significant differences in odds of new AD diagnosis when adjusting for Walk Score, though a marginally significant trend in that direction was present (OR=0.91 95% CI: 0.83,1.004; p=.0598).

Additional post-hoc analyses consisted of a best-fit multivariable model with multiple greenness conditions’ interaction terms and covariates, using the lowest QICu as the model selection criteria, as well as separate interaction tests for greenness conditions’ interaction with each moderator — age, sex, neighborhood income and Walk Score. For the multivariable interaction test we adjust for the five variables, age, sex, neighborhood income, Walk Score and race/ethnicity (for which we were underpowered to test as a moderator). We evaluated potential effect moderators when included simultaneously, as well as one at a time (interaction-separated) to confirm robustness in estimation of effect modification.

### Age

(1) In the best-fit model, the interaction of greenness category with age was statistically significant (β= 0.36; 95% CI: 0.21, 0.51; p<.0001). Specifically, for beneficiaries under age 75, there was a significantly reduced odds of developing AD in the High-High greenness compared to the Low-Low greenness condition (OR=0.68; 95% CI: 0.54, 0.85; p =.0008). In contrast, for beneficiaries ages 75 and older, there was no statistically significant difference between High-High and Low-Low greenness conditions in terms of odds of AD incidence (OR=0.97, 95% CI: 0.80, 1.18, p = 0.76). (2) In the interaction-separated model, the interaction of greenness category with age was statistically significant (β= 0.36; 95% CI: 0.20, 0.51; p<.0001). Specifically, for beneficiaries under age 75, there was a statistically significant lower odds of developing AD in High-High greenness compared to Low-Low greenness (OR=0.71; 95% CI: 0.62, 0.81; p <.0001). In contrast, for beneficiaries ages 75 and older, there was not a statistically significant relationship of greenness condition to AD incidence (OR=1.01, 95% CI: 0.92, 1.12, p = 0.80). Hence, the results from both methods showed that young-old (<75) were more likely to show an association between High-High versus Low-Low greenness and reduced odds of AD incidence, whereas old-old (> 75) did not.

### Sex

In the best fit model, the interaction between greenness category and sex was not included because the model fit improved when this interaction term was excluded. Consistent with this finding, in the interaction-separated model, the interaction of greenness category with sex was not statistically significant (β= 0.05; 95% CI: −0.10, 0.21; p = 0.495).

### Neighborhood Income

(1) In the best-fit model, the interaction of greenness category with neighborhood income approached but failed to reach statistical significance (β= 0.18; 95% CI: −0.01, 0.37; p = 0.0686). Specifically, based on a median split, for beneficiaries residing in low-income neighborhoods, there was a statistically significant reduced odds of AD incidence in High-High greenness compared to Low-Low greenness (OR=0.68; 95% CI: 0.54, 0.85; p = 0.0008), while there was a somewhat attenuated association of High-High greenness vs. Low-Low greenness to AD incidence for beneficiaries residing in higher-income neighborhoods. (OR=0.81; 95% CI: 0.69, 0.95; p = 0.01). (2) In the interaction-separated model, the interaction of greenness category with neighborhood income was statistically significant (β= 0.19; 95% CI: 0.003, 0.39; p = 0.047). Specifically, based on a median split, beneficiaries residing in low-income neighborhoods showed a statistically significant reduced odds of AD incidence in High-High greenness compared to Low-Low greenness (OR=0.78; 95% CI: 0.66, 0.92; p = 0.004). In contrast, there was no association of greenness condition to odds of AD incidence for beneficiaries residing in higher income neighborhoods (OR=0.95; 95% CI: 0.85, 1.05; p = 0.28). Hence, the results consistently demonstrated that in low-income neighborhoods there was a stronger association between greenness condition and the odds of AD incidence, whereas the association was not nearly as strong in high-income neighborhoods.

### Walk Score

(1) In the best-fit model, the interaction of greenness category with Walk Score was statistically significant (β= −0.17; 95% CI: −0.34, −0.003; p=.046). Specifically, for beneficiaries in Car Dependent neighborhoods (Walk Score 0–49), there was a statistically significant association between greenness condition and the odds of developing AD in the expected direction (OR=0.68; 95% CI: 0.54, 0.85; p=0.0008). In contrast, for beneficiaries in non-Car Dependent neighborhoods (Walk Score 50–100), there was an even stronger association between greenness condition and odds of AD incidence (OR=0.57; 95% CI: 0.46, 0.71; p<.0001). (2) In the interaction-separated model, the interaction of greenness category with Walk Score was statistically significant (β= −0.18; 95% CI: −0.34, −0.009; p = 0.04). Specifically, for beneficiaries in Car Dependent neighborhoods (Walk Score 0–49), there was no statistically significant difference between greenness conditions and the odds of developing AD (OR=0.99; 95% CI: 0.87, 1.11; p = 0.82). In contrast, for beneficiaries in non-Car Dependent neighborhoods (Walk Score 50–100), there was a statistically significant association between greenness condition and odds of AD incidence in the expected direction (OR=0.83; 95% CI: 0.73, 0.93; p=.002). Hence, there was a consistent association of high greenness to lower AD incidence in non-Car Dependent neighborhoods, but the results were inconclusive for Car-Dependent neighborhoods.

## Discussion

Higher levels of greenness have been linked with lower risk for neurological disorders such as Alzheimer’s disease, Parkinson’s disease, dementia, and stroke (9, 15–17). There is however relatively less longitudinal research evaluating the relationship between greenness and Alzheimer’s disease (14, 49). The results of this longitudinal study demonstrate that consistently higher levels of greenness over a 5-year period are associated with 16% lower odds of a new Alzheimer’s disease condition (p<.002), supporting previous cross-sectional findings (9, 10).

Further analyses explored the moderating role of age, sex, neighborhood income and walkability. Fortunately, the results were consistent across two methods: the best fit model with multiple interactions simultaneously, and the interaction separated model in which single interactions were analyzed. Those residents who showed stronger associations of high greenness to reduced AD incidence were residents aged 65–74 (young-old), in lower-income neighborhoods, and in non-Car Dependent (i.e., more urban) neighborhoods (e.g., score of >49 in Walk Score reflecting moderate to high walkability). This suggests that young-old adults residing in lower-income neighborhoods and more walkable neighborhoods — both of which tend to have lower greenness – may benefit the most, from consistently higher levels of greenness. These findings suggest that there is a specificity for whom and under what conditions high greenness may have its greatest impact. Specifically, a promising strategy for reducing AD risk may be to target low-greenness blocks for greenness-promoting intervention in lower-income, non-car dependent neighborhoods and/or in Census blocks in which young-old adults reside. In fact, results from the best-fit model comparing individuals living in consistently high vs. consistently low greenness Census blocks, revealed that when all three conditions are met simultaneously — persons younger than 75 living in neighborhoods high in walkability and low in income – there was a sizeable 32% reduced odds of AD incidence (OR=0.68, 95% CI: 0.54, 0.85, p=0.0008). It is noteworthy that inner-cities tend to be lower-income, non-car dependent, low-greenness neighborhoods, with large racial/ethnic populations, and might benefit most from greening interventions.

This study is an extension of our prior cross-sectional findings which also found that only those beneficiaries aged 65–74 years may have benefited from living in consistently high greenness Census blocks (9). The finding of these two studies, may suggest that the young-old (under age 75), may benefit more from high greenness, possibly because they are less likely to be house-bound and more able to access their outdoor environment.

The present findings are also consistent with other suggestions in the literature that residents of lower-income neighborhoods may have less access to green areas than residents of higher-income neighborhoods, which may be one pathway leading to health disparities (50). The equigenesis hypothesis proposes that high levels of greenness might mitigate the impact on health of socioeconomic inequality (50, 51). Large scale population-based studies have shown evidence of inequalities in socioeconomic standing is associated with lower levels of greenness (50, 52, 53). In a meta-analysis of 85 studies, 94% of studies showed significant evidence of moderation by SES in the relationship of greenness to health (54). Supporting this hypothesis, a study on greenspace and health in Toronto, Canada, found that living on a block with 11 more trees/block decreased cardiometabolic conditions comparably to moving to a neighborhood with $20,000 higher median income or an age reduction of 1.4 years (51, 55). Our own prior research found that the greenness to cardiometabolic and depression relationships were 50% stronger for residents of low-income neighborhoods (10, 11). The present longitudinal research further supports the equigenesis hypothesis in that Medicare beneficiaries in lower income neighborhoods had reduced AD incidence when living in high greenness Census blocks as compared to those in low greenness Census blocks. Thus, in this study, socioeconomic inequities in health outcomes, specifically AD incidence, appear to have been reduced by consistently high greenness levels across 5 years, for the young old.

This study raises the question of how higher greenness can be focused to improve health. For example, in response to climate change, a number of tree planting initiatives are being implemented around the world (56–59). The current study suggests that if those tree planting initiatives prioritized low greenness Census blocks in low-income as well as non-Car Dependent neighborhoods in which the young-old reside, climate change initiatives may also provide a beneficial health effect to this population. Since environmental interventions such as greenness are pervasive and independent of an individual’s motivation for change, these initiatives can impact both individuals as well as populations.

Greater tree-canopy and shaded streets reduce ambient temperatures, which in warmer weather can increase the comfort of walking in the shade. This, in turn, may increase time spent outdoors, and opportunities for physical activity and social interaction. (60) Physical activity and social interaction may reduce the risk of dementia (61). This is consistent with findings from Finnish Geriatric Intervention Study to Prevent Cognitive Impairment and Disability (FINGER) that revealed a causal link from exercise and social activity to improved cognitive function among older adults (6). Higher greenness may also mitigate stress through the hypothesized restorative effects of nature exposure (62). Moreover, Astell-Burt et. al. also provide support for physical activity, psychological distress, social support, sleep duration and diabetes as mediators between tree canopy and dementia (26). In addition, vegetative presence may increase exposure to diverse microorganisms that may be beneficial to the microbiome, which may have health (and possibly mental health) benefits, potentially by decreasing the risk of inflammation-induced conditions and their symptom severity (63). Other research has proposed that pollution might play a role as a mediator on the effects of greenness on health (15, 64–66).

### Limitations

The generalizability of these findings is yet to be determined. This study investigated greenness’ relationship to AD among a single large population-based sample of older adults— Medicare beneficiaries, living at least for 5 years in Miami-Dade County, FL, USA, requiring replications in other geographic areas and populations. Our research was limited to a single five-year period. Interestingly, one study in the literature reported that the impact of a tree planting intervention on mortality, required a minimum of five years after the trees were planted for a health impact to be detected (67).

Because the current study only included presence or absence of AD according to the CMS Chronic Conditions Algorithm for AD, future research might further strengthen the evidence base by longitudinally examining greenness in relation to a more detailed account of AD, its development, and progression, both behavioral (e.g., functional limitations) and biological (e.g., biomarkers, brain structure and function). It is possible that among the old-old, while greenness may not have impacted AD incidence, it could influence progression.

Medicare data did not provide systematic information on potential mediating variables such as physical activity, sleep, social interactions, or attention restoration. Moreover, potential moderators such as perceived safety or aesthetics, and genetic risk profiles (e.g., apolipoprotein E) were not available, nor were individual-level confounders such as smoking and individual socioeconomic status. We were also not able to assess the relationship of greenness to other related diagnoses such as mild cognitive impairment. Moreover, NDVI does not reveal types of greenery or vegetation. Future research might also consider alternative environmental measures of greenness as well as measures of sun exposure, humidity, and ambient temperature to better understand the relationship between health and the environment.

In addition, we were not able to assess the role of public/accessible versus private/inaccessible green areas, or access to local parks and green spaces with well-maintained infrastructure (e.g., benches and paths). We acknowledge that more-affluent versus less-affluent neighborhoods may vary in their access and/or proximity to high-quality public green spaces. Studies are therefore needed that examine the specific types and quality of vegetation and green spaces which may give us a clearer understanding of the basis for the greenness-to-AD relationship, and aid in developing possible interventions for low-income neighborhoods (e.g., canopy-preservation, tree-planting and increasing access to well-maintained parks).

## Conclusions

This study builds on prior findings suggesting that higher levels of greenness are associated with reduced odds of AD (9, 11). In the current prospective longitudinal study using a population-based sample of 230,738 U.S. Medicare beneficiaries, we found that consistently higher greenness at the Census block-level, the smallest Census geographic area available, was associated with 16% reduced odds of AD incidence when compared to consistently low block-level greenness.

These findings are consistent with prior research suggesting that greenness exposure may increase opportunities for socializing, walking, or stress mitigation that may in turn contribute to better physical and mental wellbeing in older adults (68, 69). There are two major contributions of this study: demonstrating that such as small geographic area may be associated with 16% reduced odds of AD incidence; and suggesting specificity of “for whom and under what conditions” block-level greenness is most strongly associated with reduced AD incidence. Specifically, these results suggest that young-old living in low-income and high walkability neighborhoods may show the greatest benefit in lower odds of AD incidence when residing in high (vs. low) greenness blocks.

While a person’s built and/or natural environment exposures have not traditionally been considered a modifiable risk factor in relation to AD, this study adds to the evidence-base suggesting that block level vegetation may be an important modifiable factor for reducing the odds of AD incidence.

## Notes

*Conflicts:* None.

## Notes

*Ethical standards:* Waiver of informed consent was obtained from both the UM IRB and CMS’ Privacy Board. This study was approved by the University of Miami’s Institutional Review Board (Protocols #20110948 and #20180260) and CMS’ Privacy Board (CMS DUA# RSCH-2013-24971 and CMS DUA# RSCH-2019-54747).
